# How HIV-1 Takes Advantage of the Cytoskeleton during Replication and Cell-to-Cell Transmission

**DOI:** 10.3390/v3091757

**Published:** 2011-09-15

**Authors:** Martin Lehmann, Damjan S. Nikolic, Vincent Piguet

**Affiliations:** 1 Department of Microbiology and Molecular Medicine, University Hospital and Medical School of Geneva, Geneva 1211, Switzerland; E-Mails: martin.lehmann@unige.ch (M.L.); damjan.nikolic@hcuge.ch (D.S.N); 2 Department of Dermatology and Venereology, University Hospital and Medical School of Geneva, Geneva 1211, Switzerland; 3 Department of Dermatology and Wound Healing, Cardiff University School of Medicine and University Hospital of Wales, Cardiff, Wales, CF144XN, UK

**Keywords:** HIV-1, actin, microtubules, virological synapse, dendritic cells, cell-to-cell transfer

## Abstract

Human immunodeficiency virus 1 (HIV-1) infects T cells, macrophages and dendritic cells and can manipulate their cytoskeleton structures at multiple steps during its replication cycle. Based on pharmacological and genetic targeting of cytoskeleton modulators, new imaging approaches and primary cell culture models, important roles for actin and microtubules during entry and cell-to-cell transfer have been established. Virological synapses and actin-containing membrane extensions can mediate HIV-1 transfer from dendritic cells or macrophage cells to T cells and between T cells. We will review the role of the cytoskeleton in HIV-1 entry, cellular trafficking and cell-to-cell transfer between primary cells.

## 1. Introduction

The cytoskeleton supports mechanical stability of the cell, enables its movement, division and intracellular organelle transport by way of three types of filaments: intermediate filaments, actin and microtubules. Intermediate filaments form dynamic cellular networks that provide mechanical stability and position intracellular organelles [1]. Monomers of microtubules and actin assemble into polar filaments that can be used as tracks by plus and minus end motor proteins. Kinesins and dynein are microtubule motors that mediate long range outward and inward transport, respectively. On the other hand, short range transport at the cell periphery involves myosin motor proteins moving on actin filaments [2].

For viruses that enter the cytoplasm through fusion at the plasma membrane, the cytoskeleton can represent an additional mechanical barrier. Subsequent diffusion of subviral particles larger than 500 kDa is restricted in the cytoplasm, so host cytoskeleton-associated motor proteins can be used for active transport of the genome to the nucleus for replication [3]. Transport of Adenovirus and Herpes simplex virus genomes to the nucleus relies on active transport on microtubules [4]. To efficiently infect neurons, Herpes simplex virus uses kinesins and dynein to move along the microtubules within dendrites or axons and spread through neuronal synapses [3,5]. At late stages of infection, Vaccinia virus induces actin tails that efficiently propel enveloped viral particles towards uninfected cells [6]. Furthermore, viruses can use alternative mechanisms, such as actin-rich cell surface structures and cell-to-cell contacts, for efficient viral dissemination [7].

Human immunodeficiency virus 1 (HIV-1) infects cells of the immune system, namely CD4 T cells, macrophages and dendritic cells (DCs). In order to efficiently enter these cells, HIV-1 has to recruit its fusion receptors, overcome the cortical actin cytoskeleton and transport its genome to the nucleus. Immune cells are mobile and exchange information during antigen presentation via cellular contacts, notably immunological synapses. Similarly, HIV-1 can efficiently spread between immune cells using close cell-contacts called virological synapses (VS) [8].

Here we will discuss how HIV-1 manipulates the cytoskeleton during entry, replication and cell-to-cell transmission using VS and transfer on membrane extensions. Whenever possible we will focus on data from most relevant primary cells.

## 2. The Role of the Cytoskeleton in HIV-1 Replication

### 2.1. Entry

**Figure 1.**
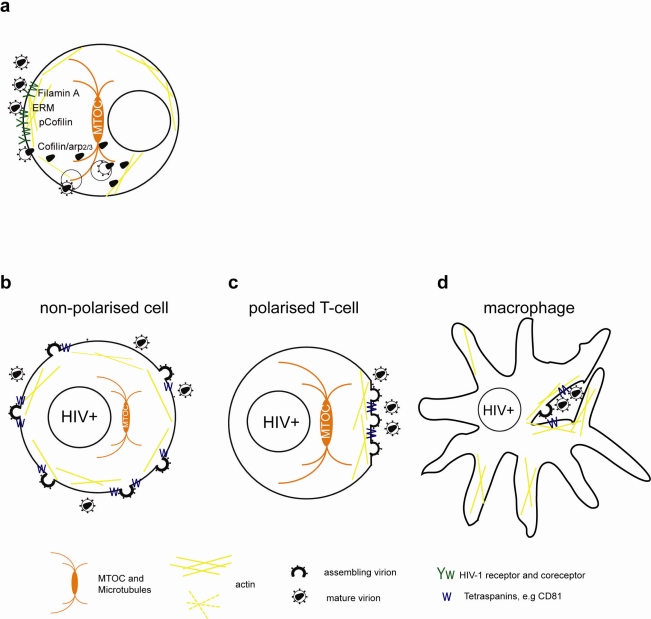
Simplified model of the role of the cytoskeleton in HIV-1 entry and release. (**a**) Binding of HIV-1 Env to CD4/CXCR4 and signaling leads to transient actin polymerization and receptor clustering through Filamin-A dependent crosslinking of CD4/CXCR4, activation of the ezrin/radixin/moesin (ERM) complex and inactivation of Cofilin. Subsequent actin depolymerization and fusion pore formation requires CXCR4 signaling to activate cofilin and Arp2/3. Viral cores can be transported on microtubules or actin towards the nucleus. Alternatively, HIV-1 enters through endocytosis and fuses with intracellular vesicles. (**b**) Usually HIV-1 assembles at the plasma membrane of non-polarized cells independently of the cytoskeleton. Note that transport of Env through the secretory pathway is not depicted. (**c**) Assembly of HIV-1 in polarized T cells occurs at actin rich pseudo-pod structures with a polarized microtubule organizing center (MTOC). (**d**) Assembly in chronically infected macrophages occurs at invaginated regions of the plasma membrane enriched in tetraspanins which are stabilized by the actin cytoskeleton.

To initiate viral membrane fusion the gp120 part of the HIV-1 envelope (Env) interacts sequentially with the primary receptor CD4 and coreceptor CXCR4 or CCR5, which leads to the exposure of the fusion promoting peptide of the gp41 part of Env [9]. Early studies indicated that the binding of HIV-1 Env to CD4 induces clustering of CD4 and CXCR4. CD4/CXCR4 clustering was dependent on actin polymerization and is required for entry and infection [10]. Similar dependency on the actin cytoskeleton was more recently described for CCR5 clustering [11]. Only recently it was shown that Env-dependent actin remodeling involves the actin-crosslinker Filamin-A, Rho-A and Rac guanosinetriphosphatases (GTPase) and actin-depolymerization factor cofilin (Figure 1). The actin-crosslinking protein Filamin-A binds the cytoplasmic tails of CD4 and CXCR4 and subsequent Env-dependent signaling leads to the activation of Rho-A [11] and Rac1 [12]. Both Rho-A and Rac GTPase activate LIM domain kinase, which phosphorylates and inactivates cofilin. Inactive cofilin triggers early actin polymerization and receptor clustering [11,12]. In addition, Env-binding to CD4 activates moesin of the ezrin/radixin/moesin (ERM) complex that promotes CD4/CXCR4 clustering [13]. Active ERM complex tethers transmembrane and cytoplasmic proteins to filamentous actin (F-actin) and promotes its membrane recruitment.

While recruitment of sufficient receptor/coreceptor complexes through actin polymerization is required to initiate fusion, the cortical actin—although a highly dynamic network—could pose a barrier to fusion pore enlargement and the passage of the capsid into the cytoplasm [14–16]. Env binding to CXCR4 induces signaling and promotes actin remodeling critical for viral intracellular entry. Activation of cofilin, an actin depolymerization factor, is required to overcome the cortical actin barrier in resting T cells [14]. Furthermore, Env signaling through CXCR4 activates Rac1-GTPase, which promotes fusion pore formation and entry via Wave2 and the actin-nucleation factor Arp2/3 [16–18].

The precise recruitment and activation of the different actin remodeling factors upon Env signaling via CD4 and coreceptor remains to be determined. Likewise, Env signaling upstream of the Rho-A/ Rac-LIMK-cofilin pathway and the kinase that phosphorylates moesin in the ERM complex have still to be ascertained. Furthermore, it is not yet clear whether Env induced receptor clustering results from centripetal actin flow generated by myosin motors or reflects a simple diffusion-capture mechanism [19].

In order to follow Env-dependent signaling and actin remodeling dynamically *in vivo*, recently developed fluorescent probes such as Rho/Rac GTPase-biosensors [20] and the fluorescent actin-probe lifeact [21] could be useful. Super-resolution microscopy can resolve and track single molecules in living cells with 20–40 nm resolution [22] and could provide detailed insight into actin-remodeling upon single virus binding events.

HIV-1 Nef, a small myristoylated protein also affects early post-fusion steps leading to increased infectivity [23,24]. The defect in infectivity of Nef-deficient HIV-1 can be complemented by disruption of the actin cytoskeleton [15] or by pseudotyping virions with Vesicular Stomatitis Virus glycoprotein (VSV-G) that fuses in low pH endocytotic vesicles.

Dynamin-2 and clathrin, both regulators of vesicular trafficking, are required in producer cells to observe the effect of Nef on infectivity [25]. By affecting clathrin-dependent endocytosis Nef could change the lipid composition of virions [25] and/or specifically remove a host factor from virions that limits fusion pore enlargement and passage of the viral core through the cortical actin cytoskeleton.

Productive infection of CD4 T cells mainly occurs through fusion at the plasma membrane [9,26]. Alternatively, HIV-1 could enter and infect cells via endocytosis and subsequent fusion with endosomal membranes [27–32]. HIV-1 may take distinct endocytotic routes in different cell types.

Clathrin-mediated endocytosis of HIV-1 was observed in HeLa cells and in primary and transformed T cells [27,28,30]. HIV-1 pseudotyped with VSV-G allows CD4/coreceptor independent entry through endocytosis in various cell types, including transformed T cells. On the contrary, infection of resting primary CD4-T cells required HIV-1 Env but not VSV-G, indicating the importance of HIV-1 Env signaling and actin remodeling in relevant primary cells [33].

Uptake into DCs requires macropinocytosis, intact actin filaments and microtubules, but their role in productive infections has not been determined [34,35]. DCs capture HIV-1 through the C-type lectin DC-SIGN and preserve infectious virions inside internal compartments enriched in actin and tetraspanins [35,36]. Conversely Langerhans cells, an epidermal subtype of DCs, capture virions via the C-type lectin langerin and efficiently degrade HIV-1 [37].

HIV-1 enters and infects macrophages following dynamin-dependent macropinocytosis, which in contrast to classical macropinocytosis, does not require myosin II and vesicle acidification [29,31].

Entry pathways in primary cells have only been characterized using a few pharmacological inhibitors that may have possible non-specific effects. Thus, the role of endocytosis in productive infection should be confirmed by genetic strategies using RNAi or dominant negative constructs. Transduction of primary macrophages and DCs by HIV-1-derived lentiviral vectors is highly effective in the presence of vpx [38].

Overall, entry through endocytosis is advantageous for the virus as it minimizes its contact with neutralizing antibodies, enables passage through the cortical actin cytoskeleton and retrograde transport towards the nucleus. Nevertheless the virus has to escape lysosomal degradation, especially in professional phagocytotic cells, like macrophages and DCs. Therefore, as recently proposed, cell type specific entry receptor levels, Env fusion kinetics and endocytosis rates can determine whether fusion occurs at the plasma-membrane or from endosomes [39].

### 2.2. Intracellular Transport

Cell fractionation has shown that the reverse transcription of viral RNA in the cytoplasm relies on interactions of the HIV-1 core with the actin cytoskeleton [40]. Live-cell microscopy revealed actin and microtubule-dependent movements of fluorescently-labeled HIV-1 [41,42]. Pharmacological inhibition of actin and microtubule polymerization arrested overall movements. Moreover, specific antibodies against dynein or genetic targeting of the dynein activator complex, dynactin, blocked minus-end transport of the particles towards the nucleus [41,42]. At the nuclear periphery, the pre-integration complex associates with perinuclear actin and eventually enters the nucleus [42]. Of note, both studies used adhered cells with a large cytoplasm and well defined microtubules. In contrast, in transformed and resting CD4 T cells microtubule integrity was not required for infection [43], indicating that short cytoplasmic distances in T cells can be overcome by actin-dependent transport. Nevertheless, microtubule-dependent transport might be necessary for the infection of adhered macrophages and dendritic cells.

Overall, it remains unclear how the viral genome is transported, uncoated and reverse transcribed and how these processes are coordinated. Notably, these steps in the HIV-1 life-cycle are sensitive to potent host restriction factors Apobec3G, TRIM5 and SAMHD1 [44–47] and their mechanisms should be better characterized.

### 2.3. Assembly and Budding

The transport of newly synthesized viral proteins towards assembly sites occurs via the secretory pathway for HIV-1 Env and via diffusion for HIV-1 Gag [9,48].

During assembly, HIV-1 Gag binds F-actin via its nucleocapsid region [49,50]. Actin, myosin and actin-binding proteins were found at assembly sites and inside virions [51–53]. During HIV-1 budding star-shaped actin structures formed when the nucleocapsid region of Gag was present [54]. Despite the interaction of actin and HIV-1 Gag, pharmacological inhibitors of actin and microtubule assembly only slightly decreased or did not affect HIV-1 release from cell lines and primary cells [10,53,55,56]. Therefore, the cytoskeleton seems not to be required for HIV-1 assembly and release from the plasma membrane of infected cells.

In polarized T cells and monocytes, HIV-1 budding was observed at polarized caps or cell protrusions (Figure 1c) [57–59]. At this actin-rich uropod structure HIV-1 Env and Gag colocalize with GM1 lipid rafts, tetraspanins and adhesion molecules [55,60–62]. Gag localization to the uropod is Env independent, but requires an intact Gag nucleocapsid part. The uropod structures copolarized with the MTOC and polarized Gag was sensitive to inhibitors of actin, myosin and microtubules, indicating a possible role of the cytoskeleton in targeted secretion and polarized budding [55,62].

In HIV-1 infected macrophages and DCs, assembly was observed on internal compartments that were subsequently identified as tetraspanin-rich sequestered plasma membrane domains [63–67]. The disruption of the actin cytoskeleton in infected macrophages decreased intracellular accumulation of HIV-1, but had no effect on the overall release [56].

Therefore, despite interaction of HIV-1 Gag with actin, there is no direct functional role of the cytoskeleton in HIV-1 assembly. Nevertheless, polarized budding at T cell uropods and intracellular budding in macrophages both depend on the cytoskeleton. The local concentration of virus at the uropod or in an intracellular compartment could facilitate subsequent cell-to-cell spread of HIV-1.

## 3. Role of Cytoskeleton in Cell-to-Cell Transfer between T Cells

### 3.1. HIV-1 Transmission

HIV-1 can spread between hosts through sexual transmission of cell-free or cell-associated virus. Major efforts are directed into the development of microbicides blocking the early steps of viral transmission at the mucosa [68]. Following entry, HIV-1 encounters potential target cells [69,70]. CD4 T cells can be directly infected in mucosal tissue [71]. Otherwise, HIV-1 can be taken up by APCs, such as dermal DCs, Langerhans cells (LC) and macrophages [69]. LC can transfer captured virus to resident T cells [71]. Alternatively, APCs can exit the mucosa and migrate to proximal lymph nodes, where captured and newly replicated virus can be transferred to T cells [70,72,73].

Cell-to-cell transfer can provide several advantages over cell free infection. (i) HIV-1 could escape detection by neutralizing antibodies, (ii) close contact limits diffusion time to a new target cell, and (iii) the VS concentrates virus and viral entry receptors to increase fusion efficiency. HIV-1 infection through cell-to-cell transfer was shown to be 100- to 18,000-times more effective than through cell-free virus [57,74]. Protection from neutralizing antibodies at the HIV-1 VS was initially observed [26,32,57], but recently challenged by another study [75]. Efficient HIV-1 cell-to-cell transfer takes place at cell-contacts called the VS, that was observed between: (i) HIV-1 pulsed DCs and T cells, (ii) infected and uninfected T cells, and (iii) infected macrophages and T cells [26,73,76,77]. Since virological synapses are cell contacts formed between different types of immune cells, analogies to antigen presentation at the immunological synapses were proposed [26,76,78].

**Figure 2.**
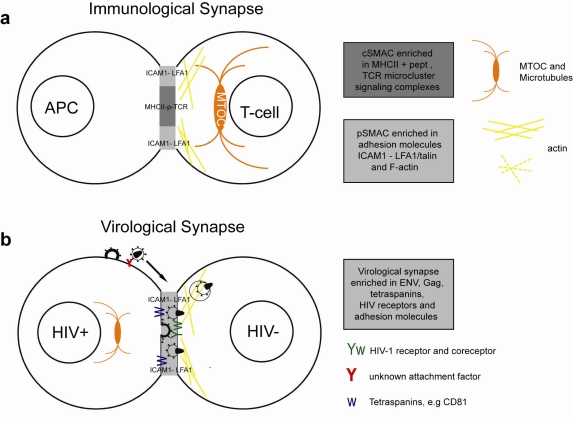

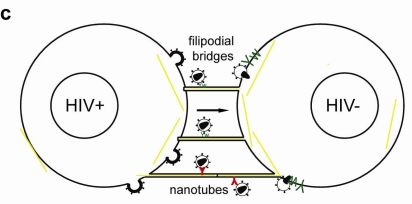
Simplified model of the immunological synapse and cell-to-cell transfer via the virological synapse or through membrane extensions (**a**) The antigen-presenting cell (APC)-T-cell immunological synapse (IS) is a stable contact. It is mediated by peptide-loaded-MHC II on the APC interacting with the TCR on the T-cell, in a central region called cSMAC, which is devoid of F-actin. Adhesion molecules ICAM1 on the APC and LFA1 on the T-cell surround and stabilize the cSMAC. In turn, talin and F-actin are organized into a peripheral ring like structure, the pSMAC. Note that costimulatory signaling through CD4/Lck, and TCR signaling leading to T-cell activation are not depicted for simplicity. (**b**) Transfer of HIV-1 between T cells occurs via the virological synapse (VS). HIV-1 Env–CD4 interactions result in clustering of CD4/CXCR4, adhesion molecules and talin at the cell contact zone, similar to the IS. HIV-1 Env signaling leads to partial T-cell activation and creates a central actin-depleted region. HIV-1 assembles directly at the VS or is transported to the contact prior to transfer. Fusion takes place directly at the target cell membrane or after endocytosis. The formation of the immunological and virological synapse are dependent on the actin and microtubule cytoskeleton (**c**) Alternatively, transfer of HIV-1 can take place on actin-containing membrane extensions: filopodial bridges or nanotubes. Filopodial bridges extend from uninfected to infected cells and transport receptor-bound virions through retrograde actin-flow. Nanotubes connect uninfected and infected cells and transport virions in an Env-independent manner.

Immunological synapses (IS) are specialized cell contacts either between antigen-presenting cells (APCs) and T cells or between target cells and effector T cells (Figure 2a). APCs can activate T cells which upon clonal expansion can execute effector functions, such as cytokine release or directed cytolysis. Peptide-major histocompatibility complex (pMHC) on the APCs engage a specific T-cell receptor (TCR), CD4 and kinases into an actin-dependent microcluster. TCR microcluster converge towards the central supramolecular activation complex (cSMAC), where TCR signaling is terminated. The cSMAC is surrounded by a ring of adhesion molecules and is associated with talin and F-actin. This peripheral SMAC (pSMAC) stabilizes the IS. The duration of the IS can be transient or last for hours. Strong pMHC-TCR affinity and costimulatory signals lead to polarization of the MTOC towards the APC and full activation of the T-cell. Cytoskeleton remodeling during T-cell activation was extensively reviewed recently [79].

### 3.2. The Virological Synapse

The structure of the VS and the mechanism of transfer are best characterized in the T cell context, but are similar in APC-T cell VS (Figure 2b).

Mobile monocytes and lymphocytes form membrane extensions at their rear, called uropods. The uropod is rich in cytoskeleton and adhesion molecules, can mediate cell-to-cell contacts and support polarized HIV-1 assembly [57–59,62]. Both polarized budding and cell-to-cell transfer depend on the actin and the microtubule cytoskeleton [26,32,55,80]. Therefore, the cytoskeleton could facilitate cell contacts, VS formation and cell-to-cell transfer through polarized budding.

The VS forms between a HIV-1 infected and uninfected cells via Env interactions with CD4 and CXCR4 or CCR5. Env and Gag concentrate on the infected cell, leading to polarized budding and transfer of virions across the VS [26,32]. On the target cell CD4 and coreceptor polarize towards the VS, thereby increasing the probability of virus fusion and the strength of the cell contact.

Additional stability to the virological synapse is conferred by adhesion molecules. Intercellular adhesion molecule 1 (ICAM-1) or ICAM-3 on the infected cell interact with the integrin lymphocyte function-associated antigen 1 (LFA-1) on the target cell [81]. Talin, an actin-bridging molecule, interacts with LFA-1 and thereby reorganizes F-actin in the target cell. Adhesion molecules, talin and F-actin form a stable ring-like structure, resembling the pSMAC [26,80,81]. Pharmacological inhibition of actin remodeling and myosin-dependent transport in target cells inhibited CD4, CXCR4 and Env clustering and transfer of infection at the VS [26,57].

Recently, live-cell imaging of CD4 T cells interacting with planar lipid bilayers containing HIV-1 Env and ICAM-1 provided a detailed structural and dynamic view of the VS [78,82,83]. Upon contact with CD4 T cells, Env forms an actin-dependent microcluster. The microclusters converge into the cSMAC and reorganize CD4 on the target cell. The CD4/Env containing cSMAC, surrounded by a ring of adhesion molecules and F-actin, transiently arrests T-cell migration [83]. Furthermore, Env induces partial TCR signaling that activates Lck and creates an F-actin depleted zone at the cSMAC [82]. Notably, some TCR recruitment and F-actin depletion from the center of the VS were also apparent in authentic T-cell contacts [26]. However, the absence of Zap70, the key mediator of TCR signaling, in target T cells does not affect HIV-1 transfer [82]. Therefore, it remains unclear if TCR signaling is required for VS formation and how Lck activation leads to depletion of F-actin from the central zone of the VS.

Alternatively, actin remodeling during the formation of the VS could also implicate Filamin-A or Env-dependent activation of ERM, Arp2/3 and cofilin as discussed before [19]. Env expressing cells induce Filamin-A dependent clustering of CD4 and CXCR4/CCR5 on target cells [11]. The role of Filamin-A, Lck, ERM, Arp2/3 and cofilin in Env-dependent signaling and actin remodeling in authentic VS needs further detailed investigation. The simplified lipid-bilayer system of the VS combined with high-resolution live-cell microscopy [84] or super-resolution techniques [22,85] could answer these questions and reveal how actin is dynamically remodeled.

HIV-1 transfer through VS relies on intact microtubules [55,80]. During transfer the microtubule organizing centre (MTOC) in HIV-1 infected T cells, but not in target cells polarizes towards the VS [7,82,86]. As expected, in infected T cells that engage multiple target cells through polysynapses the MTOC polarizes only to an individual synapse [80]. Zap70, a key mediator of TCR signaling and MTOC polarization, is required for VS formation and HIV-1 transfer. HIV-1 infected donor cells lacking Zap70 show defects in MTOC polarization and VS formation, but release normal amounts of infectious virus and establish conjugates with uninfected cells [86]. Therefore, the microtubule network may be involved in targeted secretion of viral components, adhesion molecules, tetraspanins or specific lipids to the VS that favor directed budding towards the target cell [87]. Once a stable VS is formed, the MTOC could initiate additional cell contacts leading to polysynapses.

Alternatively, polarized budding at the VS could result from incorporation of locally concentrated Env in virus particles that depend on the cytoplasmic tail (CT) of HIV-1 Env [9]. HIV-1 Env lacking the CT increases cell-to-cell transfer, but is more neutralization-sensitive than full-length Env [57]. Mouse Leukemia virus (MLV) Env concentrates at the cell-to-cell contact through interaction with receptors, interacts with MLV Gag via its cytoplasmic tail (CT) and could initiate polarized budding [88,89]. How the cytoskeleton and HIV-1 Env polarization affects polarized budding at the VS remains an important question for the future.

Dynamic imaging of the T-cell VS revealed HIV-1 Gag in button-shaped discs or ring like structures with Gag patches migrating into the synapse and transfer of viral aggregates [32,80]. Therefore, VS can provide stable contacts lasting minutes to hours that would allow polarized budding at the contact and transfer of virus from distal assembly sites. The attachment factor and details of HIV-1 movement on infected cells prior to transfer remain to be determined [89,90].

### 3.3. Actin-Containing Membrane Protrusions

Besides VS actin-containing membrane extensions, filopodial bridges, nanotubes and tunneling nanotubes have been implicated in cell-to-cell transfer of HIV-1 (Figure 2c) and other retroviruses [80,91–93]. Filopodial bridges extend from uninfected to infected epithelial cells, are stabilized through Env-receptor interaction and mediate viral transfer through retrograde actin flow towards the uninfected cell [91]. Nanotubes, on the other hand, are formed upon transient contact between T cells and connect cells over several cell lengths in curved paths. HIV-1 infection did not affect the frequency nor the direction of intercellular connections through nanotubes, but infection of target cells occurred in a receptor-dependent manner [92]. Tunneling nanotubes, however, are induced by HIV-1 infection of macrophages and contain HIV-1 particles [93].

Recently, the relative contributions of virological synapses, nanotubes and filopodial bridges to HIV-1 transfer between T cells were quantified. The authors found less than 10% of transfer occurring on nanotubes or filopodia with main contributions to viral transfer made by virological synapses [80]. Another study found 30–50% of T cells connected through nanotubes and approximately half of these structures were associated with HIV-1 Gag [92].

Live-cell microscopy visualized transfer of viral particles on nanotubes occurred with 0.08 μm/s, on filopodial bridges with 0.01 μm/s and into VS with 0.1–0.25 μm/s [91,92]. The different movement of viral particles can reflect diffusion, drift, confinements or use of distinct motor proteins and interaction with the underlying cytoskeleton in different cells [90]. Alternatively, the proximity of filopodia bridges to the glass surface and 3D matrix used to stabilize nanotubes may affect the viral movements. Therefore, the relative contributions of different cell-to-cell transfer mechanisms should be carefully examined under *in vivo* conditions in lymphatic tissue. Of note, clusters of SIV-positive cells were found in vaginal and lymph node tissue of macaques, indicating effective transfer between T cells, albeit the sensitivity and resolution of this technique limit detailed interpretation [80].

HIV-1 Nef was shown to remodel the actin cytoskeleton in infected lymphocytes leading to impaired migration in response to cytokines, inhibition of membrane ruffles and induction of filopodia-like protrusions [94,95] and reviewed in [96]. Overall reduced mobility and protrusions could enhance cell-to-cell transfer. Conversely, HIV-1 Nef modestly increased cell-to-cell transfer in lymphocytes without affecting actin remodeling in virological synapses and nanotubes [97]. Increased cell-to-cell transfer was attributed to a positive effect of Nef on infectivity [97].

Similarly, infected macrophages formed Nef-dependent long range intercellular protrusions with B cells that induced antibody class switching, leading to decreased virus-specific antibody responses [98]. The effects of Nef on cell mobility and communication between immune cells are discussed in a review by Stolp and Fackler in this issue [96].

## 4. Role of the Cytoskeleton in Virological Synapses between DCs and T Cells

DCs participate in the early HIV-1 dissemination in mucosal and lymphatic tissue [69,70,99]. Immature DCs bind, internalize and degrade extracellular material into peptides that can be presented on MHC complexes. Potential pathogens, like viruses and bacteria are recognized by pattern recognition receptors and can lead to DC maturation. During maturation DCs decrease endocytosis and upregulate chemokine-receptors and co-stimulatory proteins (CD80, CD83 and CD86). While maturating, DCs migrate to lymph nodes, where they present antigens to T cells and B cells. HIV-1 exposure does not lead to DC maturation [67,100], except when high doses of virus are used or when high infection rates are achieved in the presence of SIV vpx [101,102].

The initial maturation state of DCs upon contact has significant impact on HIV-1 infection and transfer [70,99]

While both immature DCs (iDCs) and mature DCs (mDCs) efficiently capture HIV-1, mDCs transfer virions more efficiently to T cells [73,76,103]. Alternatively, HIV-1 can infect iDCs more efficiently than mDCs and transfer newly replicated virus to T cells [70,72]. Overall, cell-to-cell transfer from DCs to T cells occurs in two phases, first transfer of captured virus occurs within 24 h after exposure and later newly replicated virus is transferred [73].

**Figure 3.**
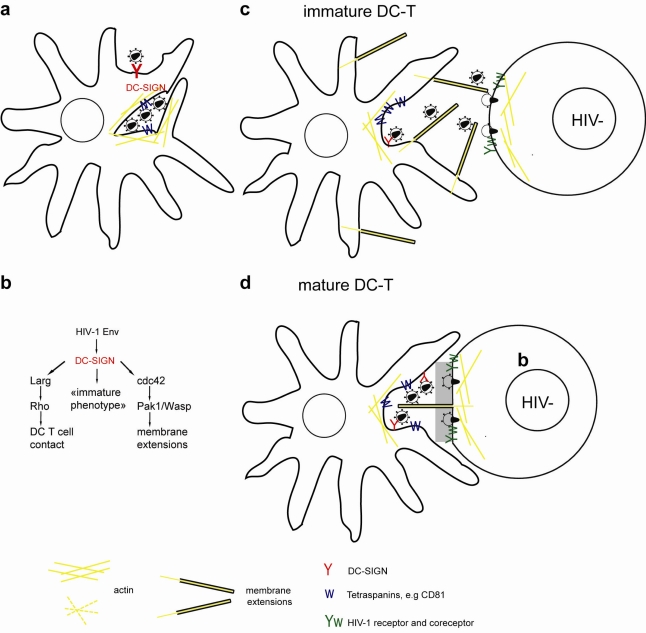
(**a**) Dendritic cells (DCs) interact with HIV-1 via DC-SIGN and capture infectious virus inside endosome-like compartments and surface accessible pockets in an actin and microtubule dependent manner. (**b**) Interaction of HIV-1 Env with DC-SIGN (i) activates Larg/Rho signaling to establish DC T-cell contacts, (ii) activates Cdc42 to induce membrane extensions, and (iii) preserves an immature DC phenotype. (**c**, **d**) Transfer of HIV-1 at DC-T cell VS occurs at cell contact zones enriched in HIV-1 receptor/coreceptor, adhesion molecules, tetraspanins and actin. (**c**) Specifically transfer from iDCs occurs on actin containing and Cdc42 dependent membrane extensions, (**d**) conversely, mDCs wrap large membrane sheets around T cells forming the virological synapse. HIV-1 was found on mDC membrane sheets and on thin membrane extensions from the T cells reaching into virus containing compartments.

Following capture, HIV-1 alters endolysosomal trafficking of DCs and localizes to surface accessible compartments that contain tetraspanins and actin, but lack MHCII (Figure 3) [35,36,67,104,105]. Upon contact with CD4 T cells a VS is formed: viral proteins and tetraspanins polarize on the DC side, while CD4, CXCR4/CCR5 and LFA-1 concentrate on the target T-cell [36,76]. Subsequent live cell imaging of the transfer from DCs and macrophages revealed dynamic translocation of HIV-1 containing compartments towards the contact zone and transfer of individual virions toward T cells [35,106,107].

Inhibition of actin remodeling in DCs pulsed with HIV-1 inhibited VS formation and transfer to T cells [34,104,107]. Therefore, actin remodeling could control trafficking of internal compartments and/or cell surface structures involved in HIV-1 DC T cell transfer.

Recent ultra-structural work revealed that HIV-1 pulsed mDCs form extensive actin-containing membrane sheets around T cells. HIV-1 localizes to internal compartments in mDCs and on membrane protrusions of T cells that reach into the virus containing compartments [104].

This analysis suggests that efficient viral transfer at the highly secluded mDC-T cell virological synapse depends on actin-containing membrane extensions. The molecular mechanisms of VS formation and actin remodeling in mDC T-cell transfer remain to be discovered.

DC-SIGN specifically binds HIV-1 through Env, localizes to the VS and is required for targeting of HIV-1 from an internal compartment to the VS and for transfer of infection [76,108].

DC-SIGN engagement with antibodies induces signaling in DC: Both ERK and Rho-GTPase, but not Rac-GTPase become activated and modulate DC maturation, cytokine release and T-cell contacts [109,110]. Specifically, HIV-1 binding to DC-SIGN activates the Rho Guanine Exchange Factor (GEF) Larg that is required for VS formation and transfer [110]. Rho-GTPases control actin-dynamics during cell migration, IS formation and were proposed to affect dendrite formation in DCs [79,110].

Recently, we used systematic siRNA knockdowns of cytoskeleton modulators like GEF, Rho-GTPases and formins in iDCs to identify pathways required for HIV-1 uptake, trafficking and transfer at the iDC-T-cell VS [107]. HIV-1 induced the formation of membrane-extensions in iDCs through Env binding to DC-SIGN and subsequent activation of the Rho GTPase Cdc42. Notably both CXCR4 and the CCR5 Env protein that mediates infection of macrophages and DCs activate Cdc42 and induce membrane extensions in iDCs. Knockdown, pharmacological inhibition and dominant-negative constructs targeting Cdc42 reduced the number of membrane extensions and HIV-1 transfer to target T cells. In iDCs fluorescent HIV-1 particles were found on dynamic membrane extensions involved in transfer across VS. Notably, when contacts between iDCs and T cells were increased by promoting IS or at high T to DC cell ratio, as found in lymph nodes, we observed that most of the HIV-1 transfer was mediated by Cdc42-dependent membrane extensions.

HIV-1 infected iDCs showed polarized budding and transfer of viral particles on dendrites towards target cells [67]. How HIV-1 infection of DCs affects actin remodeling, membrane extension and VS formation in detail remains to be determined.

## 5. Conclusions and Perspectives

During its replication cycle, HIV-1 remodels cytoskeleton structures using different viral proteins. Upon binding to cells, HIV-1 Env induces actin remodeling to concentrate receptor/coreceptors, initiate fusion and overcome the plasma membrane cortical actin network. HIV-1 entry through endocytosis and transport of its genome on actin or microtubules largely depends on the cell type. Polarized budding and cell-to-cell transfer in primary cells, but not HIV-1 assembly and release, depend on the cytoskeleton. HIV-1 uses different actin-containing structures to spread efficiently between T cells and from APCs to T cells: virological synapses, filopodial bridges, nanotubes, tunneling nanotubes and membrane extensions. A detailed mechanism for Env-induced actin remodeling at the VS is still missing. HIV-1 binding to DC-SIGN affects DC maturation, actin remodeling and induction of membrane extension in the context of DC-T cell VS.

How virological synapses and different actin-containing cell surface structures contribute to *in vivo* spread of HIV-1 remains a challenging question for the future.
